# Supplementary material for: AI-Supported Comprehensive Detection and Quantification of Biomarkers of Subclinical Widespread Diseases at Chest CT for Preventive Medicine
Source: Healthcare (Basel). 2022 Oct 29;10(11):2166. doi: 10.3390/healthcare10112166 (PMC9690402; doi:10.3390/healthcare10112166)
Supplement: Supplementary file 1 [file healthcare-10-02166-s001.zip › Figure S1 TAVI Patient.pdf]

Komorbidom Report

Patient name	Patient ID	Age	Sex	Exame date	Scanner	Slice thickness	Contrast	Kernel	Prescribing Physician
TAVI2020	TAVI2020	075Y	M	2020-12-08	Philips	3	AORTA_WF_ART_TRA	IMR1	

1 Lung analysis

1.1 Nodule detection

This analysis was done by nnDetection [1].

Number of lung nodules detected: **1**

Results for the biggest nodule:

Features	Result	Risk level
Volume	13.48mm^3	
Probability	26%	
Ellipsoid diameter per axis	1.6 (x); 3.5 (y); 4.6 (z)	

Auxiliary images

Summary:
Lung-RADS grade 2 (Benign appearance)
Recommendations:
Continue annual screening with LDCT in 12 months

This is an artificial inteligence tool for scientific purposes only.

1.2 COPD

This analysis was done by YACTA [2].

Parameters	Result	Risk level
Emphysema index (%)	6	
Bronchial wall thickning (Pi10)	0.26	

Auxiliary images

Summary:
No emphysema.
Possible bronchial wall thickening.
No bronchiectasis detected.
Recommendations:

Notes
If a condition is flagged as yellow or red, referral to an internal specialist and/or pneumologist is advised to evaluate clinical symptoms as well as possible causes and treatment.
Measurements may not be possible or false due to:
1. Network-based problems
2. Pathologies in the thorax/lungs/airways which may affect ventilation or bronchial lumen (e.g. situs after thoracotomy/resection, consolidations, neoplasm, pleural effusion, mucoid impaction).
For further reading, refer to [3].

Disclaimer
On CT images we are unable to differentiate between real bronchial wall thickening and additional wall adherent mucoid impaction. COPD phenotyping is only available with paired expiratory scan.

Abbreviation:
PRM: Parametric Response Mapping
Complete results can be found in Appendixes.

2 Bone analysis

2.1 Bone mineral density

Vertebra	Density (t-score)	Risk level
L1	85 mg/cm³ (-3.4)	
L2	98 mg/cm³ (-2.9)	
L3	100 mg/cm³ (-2.8)	
L4	114 mg/cm³ (-2.3)	
L5	100 mg/cm³ (-2.8)	
T10	110 mg/cm³ (-2.5)	
T11	99 mg/cm³ (-2.8)	
T12	88 mg/cm³ (-3.3)	

Auxiliary images

Summary:
Vertebral volume measurements not within normal range.
Recommendations:
DXA measurement recommended for suspected Osteopenia.

Notes:
Desnity is measured by mg Hydroxyapatite per cm³ of trabecular bone.

Quantification is done from TH12 to L5 if shown in the CT scan

Measurements may not be possible due to:
- Algorithm-based problems
- Pathologies in the vertebrae which may affect Bone Mineral Density (BMD) measurements (e.g. haemangiomas, vertebral fractures, bone islands, metastasis, vertebroplasty, kyphoplasty, osteosynthesis or internal fixation devices).

This is an artificial inteligence tool for scientific purposes only.

Funded by the the German state of Baden-Württemberg

3 Fat analysis

3.1 Liver and body fat

Feature	Result	Risk level
Visceral fat area (VFA)	338 cm^2	
Subcutaneous fat area (SFA)	148 cm^2	
SFA/VFA	0.44	
Intermuscular adipose tissue (IMAT)	31 cm^2	
Liver attenuation	-	
Liver fat content	-	

Auxiliary images

Summary:
Measurement of visceral and/or subcutaneous fat areas outside the range of one standard deviation.
Recommendations:
Further nutrition counselling might be beneficial.

Notes:
Severe iron overload may mask steatosis. In case of clinical suspicion, further MRI examination might be beneficial.
The formula for calculating the corresponding fat fraction is based on literature measurements with regular 120-kV scanning. This formula is not valid for different voltage settings.
The value for liver fat content is constrained to 0% for attenuation values > 65.9 HU.
For further reading, consult [4,5].

4 Vascular analysis

4.1 Cardiovascular Calcium Scoring (Agatston method)

Features	Score	Risk level
Coronary Artery Calcium (CAC)	399	
Thoracic Aortic Calcium (TAC)	12284	
Abdominal Aortic Calcium (AAC)	8214	

Auxiliary images
Summary:
Moderate CAC.
High TAC. #
High AAC.

Recommendations:
Higher risk for future cardiac events. Consider statin therapy*, and high-intensity statin therapy if Agatston score 300.*

Warning: Measurements may not be possible or inaccurate due to:
1. Algorithm-based problems;
2. Cardiovascular stents, grafts and other implants.

Notes:
* Recommendations to Coronary Artery Calcium Score: Only in adults 40 to 75 years of age without diabetes mellitus and with LDL-C levels 70 mg/dL- 189 mg/dL (1.8-4.9 mmol/L), at a 10-year ASCVD risk of 7.5% to 19.9%.
# Threshold adapted from [6] to reflect the entire thoracic aorta.
If a condition is flagged as red, referral to a cardiovascular medicine specialist is advised to evaluate possible causes and treatment.

dkfz. UNIVERSITÄTS KLINIKUM HEIDELBERG Thoraxklinik Universitätsklinikum Heidelberg
Funded by the the German state of Baden-Württemberg

5 Appendixes

YACTA complete results

Parameters	Lung	Right	Left	RUL	RML	RUL+	RLL	LUL	LLi	LUL+	LLL
Lung volume (cm^3)	4944	3457	1487	1271	493	1764	1693	1456	False	1456	31
Emphysema volume (cm^3)	301	161	141	44	36	80	80	137	False	137	4
Emphysema index (%)	6	5	9	3	7	5	5	9	False	9	14

Airways

Parameters	Mean whole tree	Lung	Right	Left	RUL	RML	RUL+	RLL	LUL	LLi	LUL+	LLL
Relative wall thickness (WP) (%) #	50	57	57	57	49	63	59	47	60	False	60	44
Pi10 *	0.26	0.26	False	False	0.4	0.23	0.38	0.1	1.1	-1.0	1.1	0.25
Bronchiectasis index (%)	False	0.08	0.12	0.0	0.38	0.0	0.25	0.02	0.0	0.0	0.0	0.0

*Standardized airway wall thickness at an internal perimeter of 10 mm.
#WP vs. 5th Generation.

Funded by the the German state of Baden-Württemberg

6 Auxiliary images

6.1 Lung nodule analysis

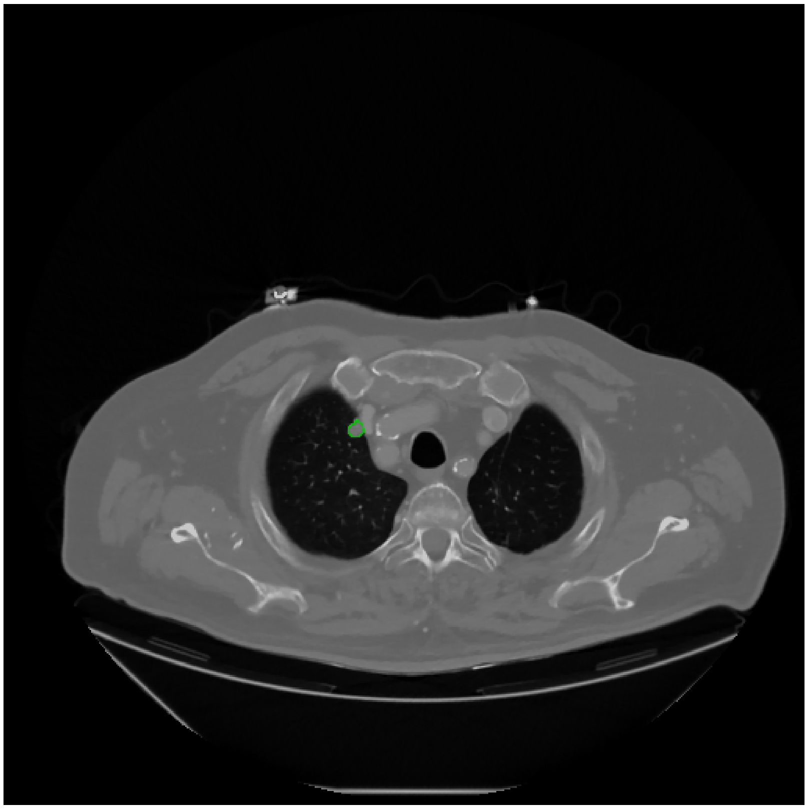

Slice number: 410

This is an artificial inteligence tool for scientific purposes only.

Funded by the the German state of Baden-Württemberg

6.2 YACTA analysis

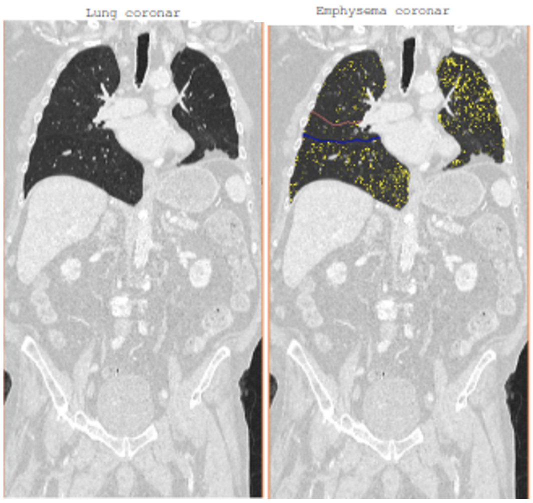

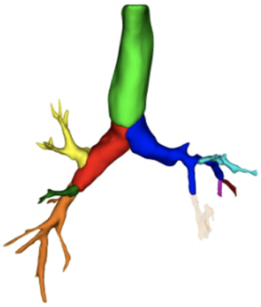

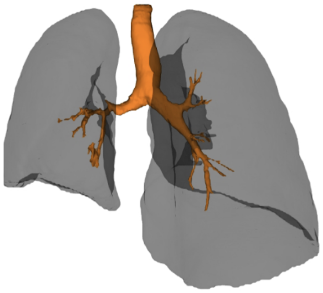

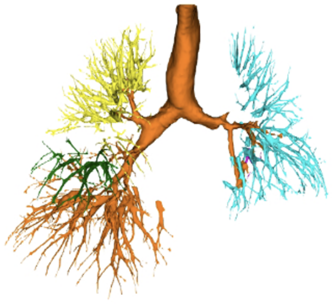

6.3 Bone analysis

Axial view at height L1 with cortical and trabecular bone extraction.

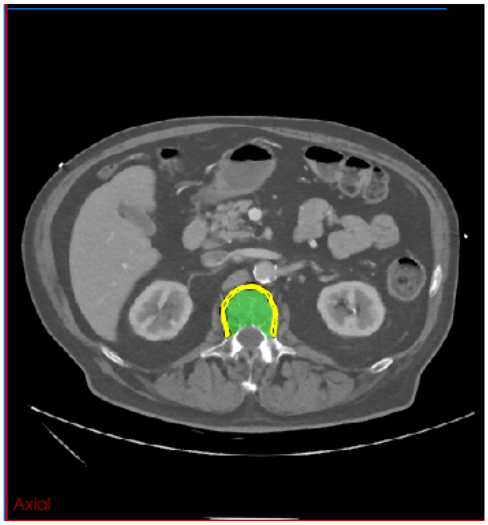

Mid-sagittal image with automated vertebrae segmentation.

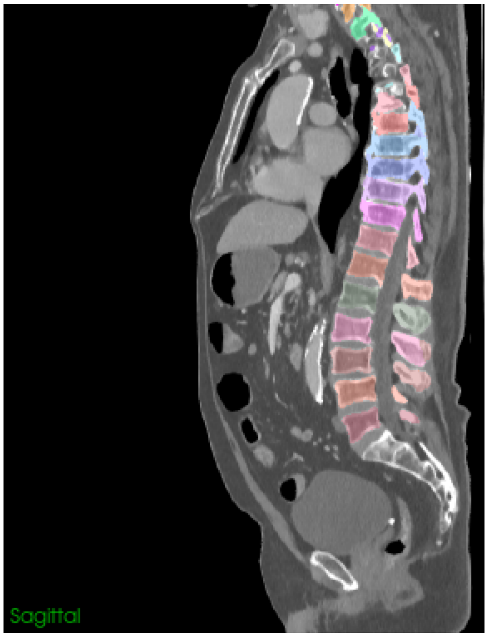

This is an artificial inteligence tool for scientific purposes only.

6.4 Fat analysis

Results for fat segmentation at lumbar intervertebral disc level L2/L3.

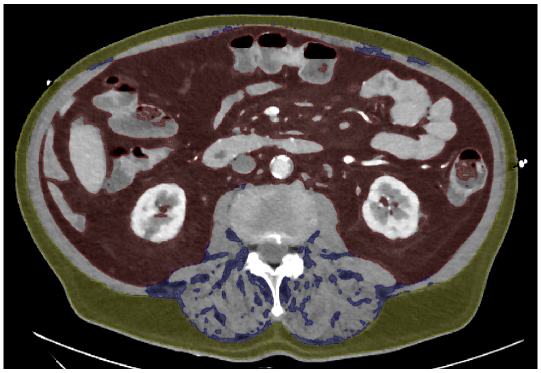

6.5 Vascular analysis

Axial (left) and sagittal (right) view of the segmentations of the coronary artery calcium (pink), the thoracic aortic calcium (blue) and abdominal aortic calcium (turquoise).

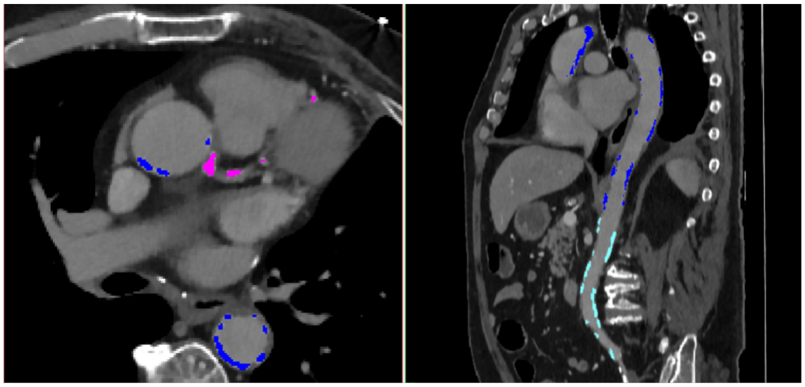

This is an artificial inteligence tool for scientific purposes only.

Funded by the the German state of Baden-Württemberg

7 References

[1] Baumgartner M., Jäger P.F., Isensee F., Maier-Hein K.H. (2021) nnDetection: A Self-configuring Method for Medical Object Detection. In: de Bruijne M. et al. (eds) Medical Image Computing and Computer Assisted Intervention – MICCAI 2021. MICCAI 2021. Lecture Notes in Computer Science, vol 12905. Springer, Cham. https://doi.org/10.1007/978-3-030-87240-3_51

[2] Heussel, C.P., Herth, F.J.F., Kappes, J. et al. Fully automatic quantitative assessment of emphysema in computed tomography: comparison with pulmonary function testing and normal values. Eur Radiol 19, 2391–2402 (2009). https://doi.org/10.1007/s003[illegible]009-1437-z

[3] Telenga ED, Oudkerk M, van Ooijen PM, Vliegenthart R, Ten Hacken NH, Postma DS, van den Berge M. Airway wall thickness on HRCT scans decreases with age and increases with smoking. BMC Pulm Med. 2017 Feb 1;17(1):27. doi: 10.1186/s12890-017-0363-0. PMID: 28143620; PMCID: PMC5286807.

[4] Irlbeck, T, Massaro JM, Bamberg F et al (2010) Association between single-slice measurements of visceral and abdominal subcutaneous adipose tissue with volumetric measurements: the Framingham Heart Study. Int J Obes (Lond) 34(4):781-787

[5] Pickhardt PJ, Graffy PM, Reeder SB (2018) Quantification of Liver Fat Content With Unenhanced MDCT: Phantom and Clinical Correlation With MRI Proton Density Fat Fraction. Am J Roentgenol 211:151-157

[6] Han D, Klein E, Friedman J et al. Prognostic significance of subtle coronary calcification in patients with zero coronary artery calcium score: From the CONFIRM registry. Atherosclerosis. 2020 Sep;309:33-38. doi: 10.1016/j.atherosclerosis.2020.07.011. Epub 2020 Jul 29. PMID: 32862086.
