# Supplementary material for: AI-Supported Comprehensive Detection and Quantification of Biomarkers of Subclinical Widespread Diseases at Chest CT for Preventive Medicine
Source: Healthcare (Basel). 2022 Oct 29;10(11):2166. doi: 10.3390/healthcare10112166 (PMC9690402; doi:10.3390/healthcare10112166)
Supplement: Supplementary file 1 [file healthcare-10-02166-s001.zip › Figure S2 COPD Patient.pdf]

Komorbidom Report

Patient name	Patient ID	Age	Sex	Exame date	Scanner	Slice thickness	Contrast	Kernel	Prescribing Physician
COPD2019	COPD2019	066Y	M	2019-07-31	SIEMENS	1	Inspiration nativ	I40f	EBERHAR_RA

1 Lung analysis

1.1 Nodule detection

This analysis was done by nnDetection [1].

Number of lung nodules detected: 0

1.2 COPD

This analysis was done by YACTA [2].

Parameters	Result	Risk level
Emphysema index (%)	33	
COPD phenotyping (%)	PRM normal: 17<br>PRM emphysema: 29<br>PRM fSAD: 52	
Bronchial wall thickning (Pi10)	0.21	

Auxiliary images

Summary:
Mild to moderate emphysema.
Possible signs of small airway disease type.
Possible bronchial wall thickening.
No bronchiectasis detected.
Recommendations:
Correlate clinical symptoms. No further intervention.

This is an artificial inteligence tool for scientific purposes only.

Funded by the the German state of Baden-Württemberg

Notes
If a condition is flagged as yellow or red, referral to an internal specialist and/or pneumologist is advised to evaluate clinical symptoms as well as possible causes and treatment.
Measurements may not be possible or false due to:
1. Network-based problems
2. Pathologies in the thorax/lungs/airways which may affect ventilation or bronchial lumen (e.g. situs after thoracotomy/resection, consolidations, neoplasm, pleural effusion, mucoid impaction).
For further reading, refer to [3].

Disclaimer
On CT images we are unable to differentiate between real bronchial wall thickening and additional wall adherent mucoid impaction. COPD phenotyping is only available with paired expiratory scan.

Abbreviation:
PRM: Parametric Response Mapping
Complete results can be found in Appendixes.

2 Bone analysis

2.1 Bone mineral density

Vertebra	Density (t-score)	Risk level
L1	52 mg/cm^3 (-4.6)	
L2	42 mg/cm^3 (-5.0)	
L3	84 mg/cm^3 (-3.4)	
L4	77 mg/cm^3 (-3.7)	
T10	70 mg/cm^3 (-3.9)	
T11	71 mg/cm^3 (-3.9)	
T12	47 mg/cm^3 (-4.8)	

Auxiliary images

Summary:
Vertebral volume measurements not within normal range.
Recommendations:
DXA measurement recommended for suspected Osteopenia.

Notes:
Desnity is measured by mg Hydroxyapatite per cm^3 of trabecular bone.

Quantification is done from TH12 to L5 if shown in the CT scan

Measurements may not be possible due to:
- Algorithm-based problems
- Pathologies in the vertebrae which may affect Bone Mineral Density (BMD) measurements (e.g. haemangiomas, vertebral fractures, bone islands, metastasis, vertebroplasty, kyphoplasty, osteosynthesis or internal fixation devices).

3 Fat analysis

3.1 Liver and body fat

Feature	Result	Risk level
Visceral fat area (VFA)	235 cm^2	
Subcutaneous fat area (SFA)	158 cm^2	
SFA/VFA	0.67	
Intermuscular adipose tissue (IMAT)	31 cm^2	
Liver attenuation	54 HU	
Liver fat content	7 %	

Auxiliary images

Summary:
Measurement of visceral and/or subcutaneous fat areas are normal.
Mild steatosis.

Recommendations:
No further recommendations for normal values of visceral and/or subcutaneous fat areas.
Follow-up with sonography recommended. Further nutrition counselling might be beneficial.

Notes:
Severe iron overload may mask steatosis. In case of clinical suspicion, further MRI examination might be beneficial.
The formula for calculating the corresponding fat fraction is based on literature measurements with regular 120-kV scanning. This formula is not valid for different voltage settings.
The value for liver fat content is constrained to 0% for attenuation values > 65.9 HU.
For further reading, consult [4,5].

This is an artificial inteligence tool for scientific purposes only.

Funded by the the German state of Baden-Württemberg

4 Vascular analysis

4.1 Cardiovascular Calcium Scoring (Agatston method)

Features	Score	Risk level
Coronary Artery Calcium (CAC)	237	
Thoracic Aortic Calcium (TAC)	2456	
Abdominal Aortic Calcium (AAC)	-	-

Auxiliary images
Summary:
Moderate CAC.
High TAC. #
No AAC.

Recommendations:
Higher risk for future cardiac events. Consider statin therapy*, and high-intensity statin therapy if Agatston score 300.*

Warning: Measurements may not be possible or inaccurate due to:
1. Algorithm-based problems;
2. Cardiovascular stents, grafts and other implants.

Notes:
* Recommendations to Coronary Artery Calcium Score: Only in adults 40 to 75 years of age without diabetes mellitus and with LDL-C levels 70 mg/dL- 189 mg/dL (1.8-4.9 mmol/L), at a 10-year ASCVD risk of 7.5% to 19.9%.
# Threshold adapted from [6] to reflect the entire thoracic aorta.
If a condition is flagged as red, referral to a cardiovascular medicine specialist is advised to evaluate possible causes and treatment.

5 Appendixes

YACTA complete results

Parameters	Lung	Right	Left	RUL	RML	RUL+	RLL	LUL	LLi	LUL+	LLL
Lung volume (cm^3)	10130	5244	4886	1764	1057	2821	2423	1458	677	2135	2751
Emphysema volume (cm^3)	3298	1409	1889	5338	291	629	779	455	231	687	1202
Emphysema index (%)	33	27	39	19	27	22	32	31	34	34	44

COPD Phenotyping: PRM % (normal / fSAD / Emphysema)*

Parameters	Lung	Right	Left	RUL	RML	RUL+	RLL	LUL	LLi	LUL+	LLL
Normal (%)	17	21	14	22	24	23	18	16	16	16	12
fSAD (%)	52	54	49	60	50	56	51	55	51	53	46
Emphysema (%)	29	23	34	16	23	19	28	27	29	28	40

*only available with paired expiratory scan

Airways

Parameters	Mean whole tree	Lung	Right	Left	RUL	RML	RUL+	RLL	LUL	LLi	LUL+	LLL
Relative wall thickness (WP) (%) #	49	50	50	50	48	58	50	54	52	46	48	54
Pi10 *	False	0.21	False	False	0.11	0.24	0.17	0.25	0.23	0.2	0.22	0.21
Bronchiectasis index (%)	False	0.52	0.43	0.66	0.13	0.0	0.08	1.06	0.0	0.0	0.0	1.37

*Standardized airway wall thickness at an internal perimeter of 10 mm.
#WP vs. 5th Generation.

This is an artificial inteligence tool for scientific purposes only.

Funded by the the German state of Baden-Württemberg

6 Auxiliary images

6.1 Lung nodule analysis

No image was generated.

6.2 YACTA analysis

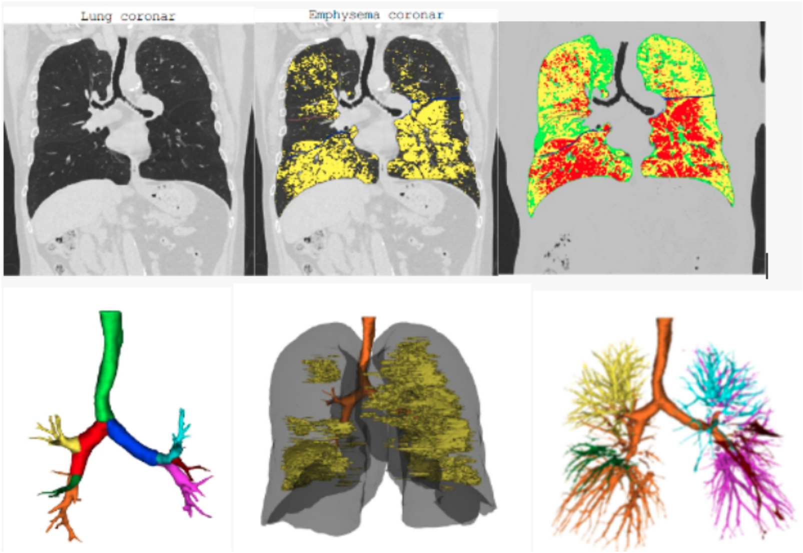

6.3 Bone analysis

Mid-sagittal image with automated vertebrae segmentation.

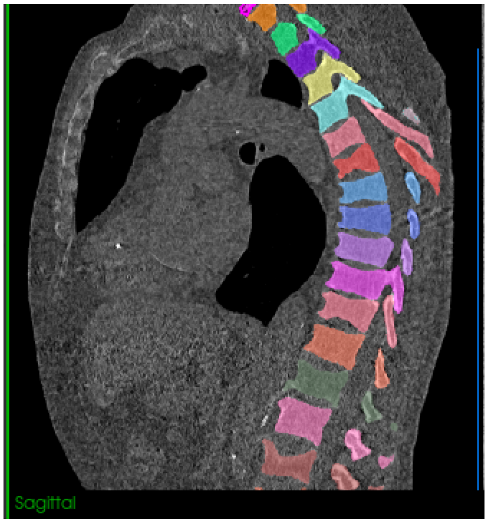

Axial view at height L1 with cortical and trabecular bone extraction.

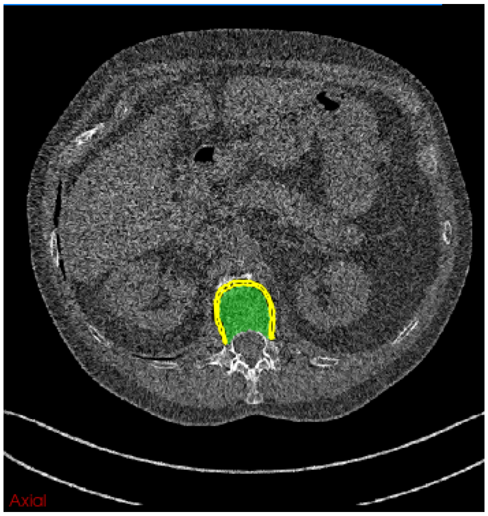

6.4 Fat analysis

Results for fat segmentation at lumbar intervertebral disc level L2/L3.

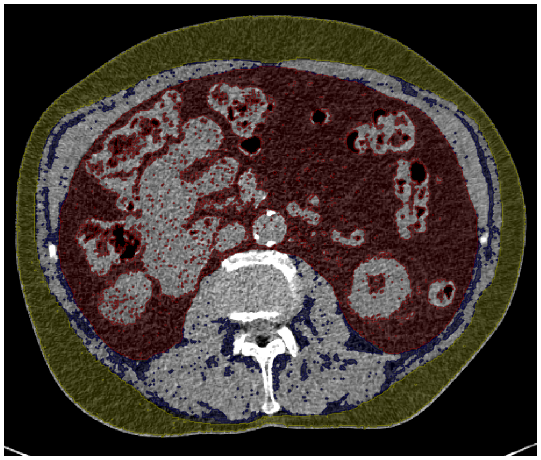

6.5 Vascular analysis

Axial (left) and sagittal (right) view of the segmentations of the coronary artery calcium (pink) and the thoracic aortic calcium (blue).

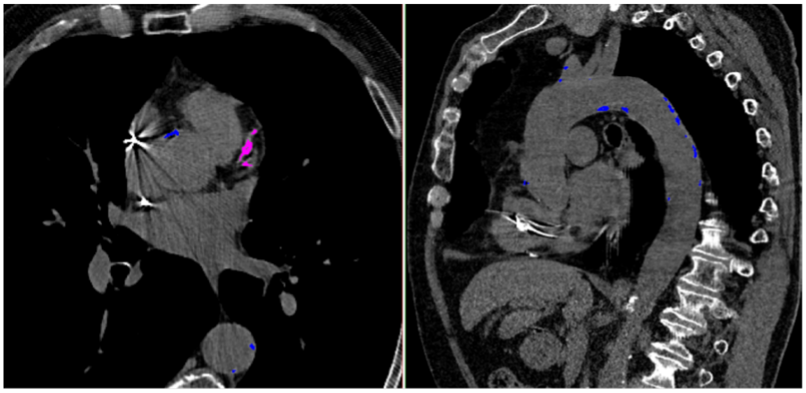

This is an artificial inteligence tool for scientific purposes only.

Funded by the the German state of Baden-Württemberg

7 References

[1] Baumgartner M., Jäger P.F., Isensee F., Maier-Hein K.H. (2021) nnDetection: A Self-configuring Method for Medical Object Detection. In: de Bruijne M. et al. (eds) Medical Image Computing and Computer Assisted Intervention – MICCAI 2021. MICCAI 2021. Lecture Notes in Computer Science, vol 12905. Springer, Cham. https://doi.org/10.1007/978-3-030-87240-3_51

[2] Heussel, C.P., Herth, F.J.F., Kappes, J. et al. Fully automatic quantitative assessment of emphysema in computed tomography: comparison with pulmonary function testing and normal values. Eur Radiol 19, 2391–2402 (2009). https://doi.org/10.1007/s003[illegible]009-1437-z

[3] Telenga ED, Oudkerk M, van Ooijen PM, Vliegenthart R, Ten Hacken NH, Postma DS, van den Berge M. Airway wall thickness on HRCT scans decreases with age and increases with smoking. BMC Pulm Med. 2017 Feb 1;17(1):27. doi: 10.1186/s12890-017-0363-0. PMID: 28143620; PMCID: PMC5286807.

[4] Irlbeck, T, Massaro JM, Bamberg F et al (2010) Association between single-slice measurements of visceral and abdominal subcutaneous adipose tissue with volumetric measurements: the Framingham Heart Study. Int J Obes (Lond) 34(4):781-787

[5] Pickhardt PJ, Graffy PM, Reeder SB (2018) Quantification of Liver Fat Content With Unenhanced MDCT: Phantom and Clinical Correlation With MRI Proton Density Fat Fraction. Am J Roentgenol 211:151-157

[6] Han D, Klein E, Friedman J et al. Prognostic significance of subtle coronary calcification in patients with zero coronary artery calcium score: From the CONFIRM registry. Atherosclerosis. 2020 Sep;309:33-38. doi: 10.1016/j.atherosclerosis.2020.07.011. Epub 2020 Jul 29. PMID: 32862086.
